# Valorization of Citrus Co-Products: Recovery of Bioactive Compounds and Application in Meat and Meat Products

**DOI:** 10.3390/plants10061069

**Published:** 2021-05-26

**Authors:** Gema Nieto, Juana Fernández-López, José A. Pérez-Álvarez, Rocío Peñalver, Gaspar Ros, Manuel Viuda-Martos

**Affiliations:** 1 Department of Food Technology, Nutrition and Food Science, Veterinary Faculty, University of Murcia, Regional Campus of International Excellence, “Campus Mare Nostrum”, Campus de Espinardo, 30100 Espinardo, Spain; gnieto@um.es (G.N.); rocio.penalver@um.es (R.P.); gros@um.es (G.R.); 2 IPOA Research Group, Agro-Food Technology Department, Centro de Investigación e Innovación Agroalimentaria y Agroambiental (CIAGRO-UMH), Miguel Hernández University, Orihuela, 03312 Alicante, Spain; j.fernandez@umh.es (J.F.-L.); ja.perez@umh.es (J.A.P.-Á.)

**Keywords:** orange, lemon, mandarin, grapefruit, co-products, polyphenolic compounds, antioxidant, antibacterial, meat products

## Abstract

Citrus fruits (orange, lemon, mandarin, and grapefruit) are one of the most extensively cultivated crops. Actually, fresh consumption far exceeds the demand and, subsequently, a great volume of the production is destined for the citrus-processing industries, which produce a huge quantity of co-products. These co-products, without proper treatment and disposal, might cause severe environmental problems. The co-products obtained from the citrus industry may be considered a very important source of high-added-value bioactive compounds that could be used in the pharmaceutical, cosmetic, and dietetic industries, and mainly in the food industry. Due to consumer demands, the food industry is exploring a new and economical source of bioactive compounds to develop novel foods with healthy properties. Thus, the aim of this review is to describe the possible benefits of citrus co-products as a source of bioactive compounds and their applications in the development of healthier meat and meat products.

## 1. Introduction

Citrus fruits (*Rutaceae* family), including orange (*Citrus sinensis*), lemon (*Citrus limon*), mandarin (*Citrus reticulata*), and grapefruit (*Citrus paradise*), are the most produced fruits worldwide, both in the Northern and Southern Hemispheres. There are two main factors that make this possible: on the one hand, their physical properties give them unbeatable qualities for conservation (meaning they can be transported to any place in the world) and, secondly, they are among the few winter fruits that grow equally in both hemispheres. Thus, worldwide, citrus production has continued to grow during the last decades. To a greater or lesser extent, the nearly ninety citrus-producing countries have increased their production volumes, giving rise to increasing numbers worldwide, even despite the strong recession that has occurred due to several diseases such as “citrus greening,” among others.

In 2019, citrus fruits had an annual production of approximately 143.48 million tons, with the main citrus-producing country being China with 38.19 million tons, followed by Brazil (19.65 million tons), India (12.99 million tons), Mexico (8.41 million tons), the United States (7.25 million tons), and Spain (6.00 million tons) [1]. Among the citrus fruits, orange production constitutes around 54.84% of the total world production followed by mandarin with 24.70%; finally, the remaining 20.44% corresponds to the sum of lemon and lime (13.97%) and grapefruit (6.47%). The great production of citrus fruits is motivated by the highly appreciated sensorial properties (odor, flavor, and taste) of these fruits as well as the nutritional quality associated with fresh consumption [2]. Currently, the production of citrus fruits for fresh consumption far exceeds the demand; consequently, a great volume of the production is destined for the food industry, mainly the juice industry. It has been estimated that 40% of citrus harvested in the world is used in the production of juices [3], although several commercial products including jams, jellies, flavoring agents for beverages, and canned products are also produced. This industrialization process generates high volumes of co-products, which, due to their chemical and physical-chemical characteristics, are very difficult to stock. Traditionally, these co-products were used for animal feeding or for compost production [4]. However, in their composition, it is possible to find several compounds with high added value that, after the appropriate conversion process, could be transformed into marketable products, either (i) as raw supplies for other processes, (ii) as manage materials, or (iii) as ingredients for new food products development, to obtain the benefit of the great amount of potentially valuable compounds that they contain [5]. For this reason, the citrus juice industry has had to implement new processes for the transformation of the biomass obtained from the squeezing of citrus fruits, with the dual purpose of reducing its environmental impact and improving the income statement through the commercialization of the co-products obtained, in what has come to be called in recent times the circular economy models of the agri-food industry [6,7].

Citrus co-products obtained from juice production are composed of two fractions such as peel and pulp (seeds and membrane residues). These fractions are a great source of several bioactive compounds including dietary fiber (pectin, cellulose, hemicellulose, lignin), minerals (potassium, calcium, magnesium, selenium), organic acids (citric, oxalic, and malic acids), vitamins (vitamin C, thiamine, riboflavin, and niacin), phenolic acids (chlorogenic, ferulic, and sinapic acids), flavonoids (hesperidin, narirutin, didymin, hesperetin, diosmin), terpenes (limonene), carotenoids (lutein, β-carotene, zeaxanthin), etc. [8,9,10,11,12,13]. These bioactive compounds have demonstrated several health effects such as antioxidant, antimicrobial, anti-inflammatory, anti-hypertensive, neuroprotective, antimutagenic, and antiallergic properties [8,14,15,16]. Thus, there is an increasing interest by the pharmaceutical, cosmetic, and food industries in using co-products from the citrus industry in the development of numerous products. In this way, the meat industry is knowledgeable about the consumer demands for healthier meat and meat products with a reduced level of fat and cholesterol, decreased contents of sodium chloride and nitrite, the improved composition of the fatty acid profile, and incorporated health-enhancing ingredients [17]. The most important actions for the development and improvement of healthier meat and meat products focus on: (i) Action on the genome based on selection and crossover. (ii) Action through animal nutrition with the use of diets where wild plants, fish oils, microalgae, etc. have been included in order to increase the content of omega-3 fatty acids. (iii) Action on the production process introducing changes in meat and non-meat ingredients [18].

This review focuses on the health benefits of citrus co-products by summarizing the current body of research focusing on citrus co-products’ bioactive compounds and their applications in the development of healthier meat and meat products.

## 2. Sustainability in the Agri-Food Industry

Among the most relevant factors that influence the consumer in the purchase act are health and sustainability. Satisfying both aspects is a competitive advantage in the market distribution. The sustainability of the agri-food industry is becoming one of the main challenges of this sector since it must take into account the growing demand of society to consume products that are not only healthy and safe but also produced under criteria of social responsibility and environmental sustainability [19]. The efficient use of the co-products that are generated during agri-industrial activity, regardless of the sector in question, is a priority for the food industry, as it could lead to environmental problems derived from their accumulation, handling, and elimination [20]. The valorization of co-products has environmental and economic benefits for the food sector, thus contributing to the creation of sustainable value chains in both the agricultural and processing sectors [21]. Currently, there is a great demand for the transformation of co-products, through sustainable processes, into new ingredients. The recovery of these co-products entails a reduction in disposal costs and an improvement in the environmental impact that, together with adequate technology, allows their transformation into products of interest for different sectors such as the food, dietetic, pharmaceutical, cosmetic, etc. industries. [22]. Likewise, sustainable agriculture must support the sustainable management of land, water, and natural resources. Agriculture must meet the needs of present and future generations for its products and services while ensuring profitability, environmental health, and social and economic equity [23]. One of the priorities of this policy is efficiency in the use of resources to promote local and regional agricultural markets.

## 3. Co-Products Associated with the Citrus Juice Extraction Processes

The principal product of the citrus processing industry is juice. In general, citrus fruits have a juice content that ranges between 45% and 58% depending on the variety. Therefore, the process of transformation of citrus fruits to obtain juices generates a great quantity of co-products (around 50% to 70% w/w of the weight of the fruit, depending on adopted technology and fruit cultivar), and its annual world production is probably close to 10 million Mg [24]. These co-products are composed of around 60–65% peel, 30–35% internal tissues, and up to 10% seeds by weight [25].

**Figure 1:**
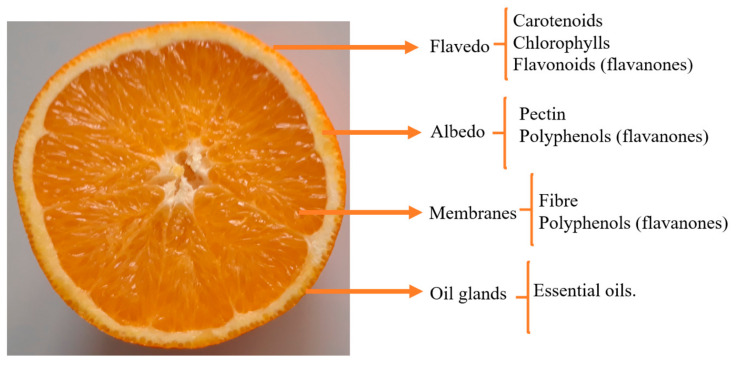
Schematic view of structural composition of citrus.

As mentioned above, these co-products are composed of two fractions, peel and pulp (seeds and membrane residues). The schematic view of the structural composition of citrus has been shown in Figure 1.

**Figure 2:**
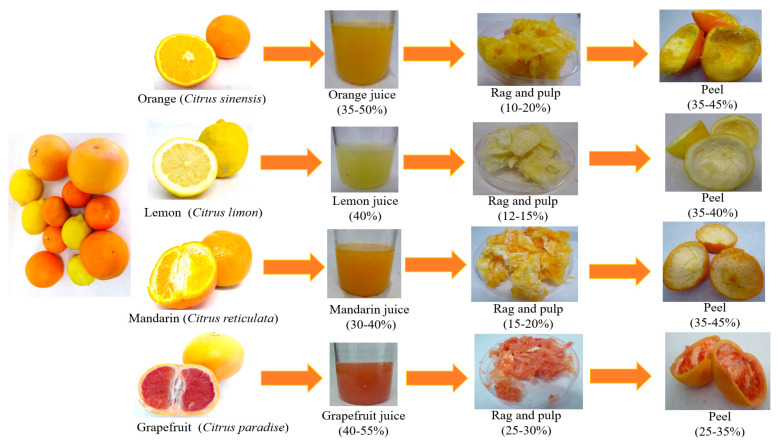
The principal citrus species and their composition in terms of juice and co-products composed by peel and rags and pulp residues.

The peel comprises flavedo and albedo. Flavedo is the external layer of the fruit, which is typically composed of cellulosic material but also contains other components, such as essential oils (monoterpenes, alcohols, aldehydes), pigments (carotenoids, chlorophylls, flavonoids), steroids and triterpenoids, and paraffin waxes [10]. The albedo is joined to the flavedo. This structure is a sponge-like white tissue, predominantly rich in pectin substances. Its thickness varies widely among the different citrus varieties, from a few millimeters in mandarins to 1–2 cm or more in grapefruit [26]. Besides pectin, in its composition it is possible to find various bioactive compounds such as vitamins and polyphenols—mainly flavanones such as hesperidin, narirutin, didymin, and diosmin [27]. Pulp or rag co-products involve the fraction screened from the pulp, being cores, segment walls or membranes, juice vesicles, and seeds. Rag and pulp are composed of dietary fiber (mainly insoluble fiber (cellulose and hemicellulose)), organic acids (citric, oxalic acids), sugars (glucose and fructose), and polyphenols (mainly flavonoids) [28]. Figure 2 shows the major citrus species and their physical composition in terms of juice and co-products composed of peel and rags and pulp residues.

## 4. Main Bioactive Compounds Present in Citrus Co-Products

A diet is not only limited to its nutrient content but also provides other compounds that protect us against oxidative stress and carcinogenesis: compounds that are mostly found in foods of plant origin [29]. One of the main sources of bioactive compounds is the vegetables and fruits. Thus, four large groups of bioactive compounds can be distinguished in the plant kingdom: (i) nitrogenous substances, (ii) sulphur substances, (iii) terpenic substances, and, the most studied, (iv) phenolic substances [30], the last two groups being the bioactive compounds mostly present in fruits [31]. In this way, citrus fruits in all their species and varieties are characterized by their high content of bioactive molecules [9]. These bioactive molecules can be divided into non-phenolic compounds and phenolic compounds.

### 4.1. Non-Polyphenolic Compounds

There are many non-phenolic bioactive compounds that can be found in citrus fruits, such as organic acids, vitamins, terpenes, and carotenoids [32,33,34,35].

Organic acids play an important role among the biologically active compounds. These acids are found in citrus fruits in high quantities and some of them have antioxidant activity. The main organic acids present in citrus fruits are citric and malic acid, with citric acid being the most important and which enhances the action of vitamin C [32]. Citric acid (found in lemon in an amount between 7 and 9%, and between 0.6 and 1.5% in orange and mandarin) is the main acid in the endocarp of all citrus fruits [36].

Vitamin C (ascorbic acid) is the most abundant vitamin in citrus fruits, which is why these fruits are the main natural source for obtaining it. The rind of citrus fruits is especially rich in this vitamin; the percentages in orange are 34% in the flavedo, 19% in the albedo, 21% in the pulp, and 26% in the juice. In grapefruit, these values are 31%, 33%, 19%, and 17%, respectively [33,37].

Other potentially beneficial bioactive molecules present in citrus fruits are carotenoids. Reynoso et al. [35] found that oranges and mandarins contain lutein, zeaxanthin, cryptoxanthin, violaxanthin, luteoxanthin, auroxanthin, and other carotenoids in both peel and pulp. Zhou et al. [38] studied the carotenoid content of eight citrus varieties and found that the peels of Ponkan mandarin, Kumquat (*Citrus microcarpa*), and Liucheng orange contained the highest amount of lutein, zeaxanthin, β-cryptoxanthin, and β-carotene, with the levels in the peel being much higher than the levels in the fruit. Murador et al. [39] reported that the main carotenoids found in peels (flavedo and albedo) of orange cv. Pera cultivated in Brazil were (all-E)-lutein, (all-E)-β-carotene, and violaxanthin.

Pectin is another bioactive compound present in citrus peels. Naturally, the pectin is methyl esterified at the carboxyl group of some of its galacturonic acid residues, based on which it is classified into two groups: high methoxyl pectin (degree of esterification, >50%) and low methoxyl pectin (degree of esterification, <50%) [40]. The pectin content that can be found in the peel of citrus fruits varies depending on the species and variety. Thus, Wang et al. [41] reported values of 86.4, 37.3, 65.2, and 43.7 mg/g peel for *Citrus grandis, C. reticulata, C. limon*, and *C. sinensis*, respectively.

Finally, the essential oil contained in the flavedo varies in a range of 0.5 to 3.0 kg/ton of orange. It is composed of terpenes (>90%), oxygenated compounds, and non-volatile compounds [34]. The main components of citrus peel essential oils include monoterpenes, sesquiterpenes, and their oxygenated derivatives. Specifically, limonene is the major oil component identified in the peel of different citrus species [42].

### 4.2. Polyphenolic Compounds

Organic polyphenolic compounds, flavonoids, and non-flavonoid phenolic compounds represent one of the most numerous and complex groups of secondary plant metabolites [43]. These molecules are one of the most widely distributed groups of substances in the plant kingdom and are divided according to the number of phenolic rings they possess and the structural elements present in these rings [44]. They comprise a wide variety of compounds divided into four distinct classes: (i) phenolic acids including hydroxybenzoic acids and hydroxycinnamic acids, (ii) flavonoids such as flavones, flavonols, flavanones, flavanonols, flavanols or catechins, anthocyanins, and chalcones, (iii) stilbenes, and (iv) lignans [45]. Citrus fruits are also a very important source of this type of biomolecules in all their forms and types [46]. Among all flavonoids found in these fruits, flavanones (narirutin, hesperidin, naringin, and neohesperidin), flavones (luteolin, apigenin, diosmin), flavonols (rutin, quercetin, kaempferol), and anthocyanins are in major quantities [4,22]. It is important to notice that phenolic compounds exist not only in comestible parts of citrus fruit but also in non-edible fractions (especially citrus peels) with multiple biological functions [11].

Phenolic acid content and composition vary among different citrus species. Hydroxycinnamic acids are a group of compounds present in the cell wall of citrus fruits in general and particularly in lemon, whose main representatives are chlorogenic acid, ferulic acid, *p*-coumaric acid, caffeic acid, and sinapic acid [47], of which ferulic and chlorogenic acid are the most abundant in these fruits [48]. Kelebek [49] also identified hydroxybenzoic acids in citrus fruits, particularly in grapefruit, with gallic acid, protocatechuic acid, p-hydroxybenzoic acid, and vinylic acid being the principal compounds.

**Figure 3:**
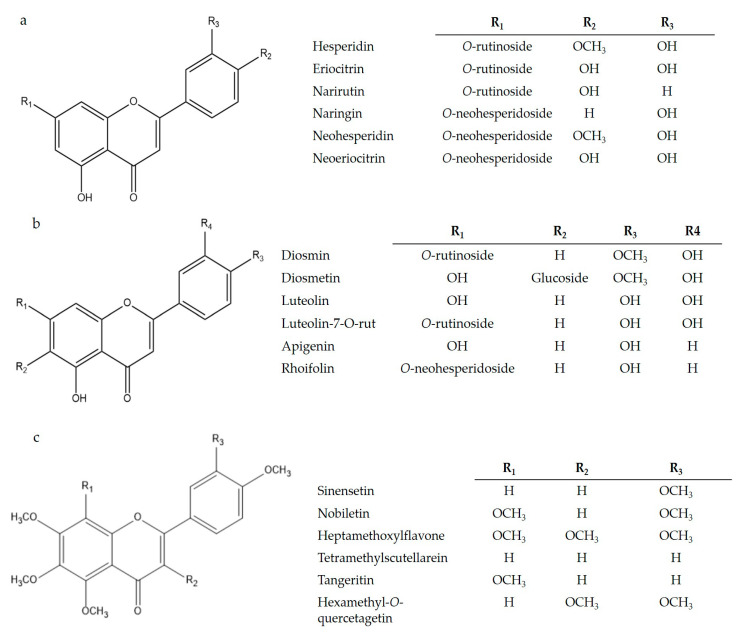
The main flavanones (**a**), flavones (**b**), and polymethoxylated flavones (**c**) found in citrus fruits. Source [51,52,53,54,55].

Flavonoids are present in most plants and constitute a very important class of bioactive compounds in citrus species; they are mostly found as glycosides, but they can also occur in free form (flavonoid aglycones) [44]. In addition, they can occur as sulphates, dimers, or polymers. Citrus glycosides can be found in two forms: as *O*-glycosides with the carbohydrates linked via oxygen atoms (hemiacetal bond), or as C-glycosides with the carbohydrates linked via carbon-carbon bonds. Of all these natural forms, *O*-glycosides are the most prevalent [45]. As mentioned above, flavanones (Figure 3a) and flavones (Figure 3b) are the main flavonoids found in the peel of citrus fruits. Within the group of flavones, receiving the generic name of polymethoxylated flavones (Figure 3c) is a chemical family of compounds that have a number of methoxyl groups equal to or greater than four. These compounds are also practically exclusive to citrus [50].

The main flavonoids found in citrus peel are glycosylated flavanones (hesperidin naringin and narirutin) located mainly in the albedo [51] and polymethoxylated flavones (sinensetin, hexamethyl ether quercetagetin, nobiletin, tetramethyl scutellarein, 3,5,6,7,8,3’,4’-heptamethoxyflavone, and tangeretin) present in the flavedo [52]. Others include glycosides flavanones (molecules with sugar residues) found in citrus peels, including poncirin, neohesperidin (hesperitin-7-neohesperidoside), didymin, neoeriocitrin (heridictyol-7-neohesperidoside), and eriocitrin [53]. On the other hand, the aglyconated flavanones (molecules not bound to sugar residues) present in citrus peels comprise naringenin (40,5,7-trihydroxyflavanone), hesperetin (3,5,7-trihydroxy-40-methoxyflavanone), isosakuranetin (3,5-dihydroxy-40-methoxyflavanone), and eriodictyol (5,7,30,40 tetrahydroxyflavone) [16,54,55].

## 5. Antioxidant and Antimicrobial Activity of Bioactive Compounds Present in Citrus Co-Products

### 5.1. Antioxidant Properties

Polyphenolic compounds act as antioxidants, as they are able to prevent oxidation. This property of the compound will depend on the oxidation-reduction of the hydroxyphenolic group and the chemical structure; however, in a fruit, the antioxidant capacity is not simply given by the sum of the antioxidant capacities of each of its components but also by the interaction between them, which can produce synergistic or antagonistic effects [56].

Bioactive compounds existing in citrus peel co-products showed high antioxidant capacity against free radicals. Antioxidant effects are the best-described property of polyphenols; while almost all flavonoids have the ability to act as antioxidants, flavones and catechins seem to have a higher activity, protecting our organism from free radicals—molecules that have an unpaired electron in their atomic structure and generate a redox reaction [11,16]. The antioxidant properties of several extracts, rich in bioactive compounds, obtained from citrus co-products were widely demonstrated [57,58,59,60,61,62,63,64,65,66] (Table 1). Nevertheless, it should be noted that antioxidant activity differs between the peels of different citrus species due, basically, to different composition in bioactive compounds as well as the methodology used to obtain the extracts and environmental conditions, including the climate, soil and irrigation practices, stage of fruit ripening, cultural practices, and time before harvest [67,68]. In this sense, Nayak et al. [48] carried out a study to analyze the antioxidant activity (using DPPH and ORAC-values) of *C. sinensis* peel extracts using a microwave-assisted extraction or ultrasound-assisted extraction. For DPPH assay, the values reported to inhibit the formation of DPPH radicals in 50% were 337.16 and 433.09 mL/L, respectively, while for ORAC assay, the values reported were 482.27 and 456.94 µMol Trolox Equivalent/g sample for microwave-assisted extraction and ultrasound-assisted extraction, respectively. Similarly, Chen et al. [51] reported that the antioxidant activity of extracts obtained from dried *C. reticulata* peels (exocarp and pericarp) cultivated in China varied in ORAC assay from 1033.8 to 1331.7 µMol Trolox Equivalent/g sample, while for DPPH, assay values reported to inhibit the formation of DPPH radicals in 50% ranged from 0.52 to 0.68 mg/mL. In a similar study, Lagha-Benamrouche and Madani analyzed [69] the antioxidant activity of peel extracts obtained for different cultivars of species *C. sinensis* and *Citrus aurantium* cultivated in Algeria. The antiradical activity values, using the DPPH assay, for examined extracts varied between 55.49 and 88% for the peels, with values reported to inhibit the formation of DPPH radicals in 50% ranging between 0.56 and 0.91 mg/mL. In a similar study, Papoutsis et al. [70] analyzed the antioxidant capacity of lemon pomace. These authors found that the aqueous or methanol extracts of lemon pomace had antioxidant activity values, measured with DPPH assay, of 0.17 and 0.13 mg Trolox equivalents/g sample, respectively, while for ABTS assay the values reported were 0.403 and 0.458 mg Trolox equivalents/g sample, respectively. Abudayeh et al. [71] analyzed the hydroalcoholic extracts obtained from pomelo (*Citrus maxima*) peels. These authors reported that the DPPH radical scavenging activity exhibits a significant dose-dependent inhibition of DPPH radical color, with values to inhibit the formation of DPPH radicals in 50% of 68.55 μg/mL of the extract, comparable to 55.87 μg/mL of vitamin C.

### 5.2. Antimicrobial Properties

Several extracts obtained from citrus co-products could contain, in their composition, several phytochemicals including polyphenolic compounds (phenolic acid and flavonoids) and carotenoids, which can exert antibacterial and antifungal activities [60,72,73,74,75,76,77,78,79,80,81,82,83,84]. Table 2 shows the antibacterial activity of peel extracts or compounds obtained from different *Citrus* species. This antibacterial activity, in some cases, characteristically results from the combination of these secondary bioactive products. Thus, polyphenolic compounds present in citrus co-products demonstrated that they can act as antimicrobial agents, i.e., they could inhibit the growth of micro-organisms, depending on the structure and content of the hydroxyl group [85]. Those polyphenolic compounds with antimicrobial activity could cause cell lysis of micro-organisms by different mechanisms of action including blocking the synthesis of the cell wall or the transport of its precursors; affecting the cytoplasmic membrane; blocking the phases of protein synthesis such as activation, initiation, and attachment of the amino acid-tRNA complex to the ribosome (and could even affect the metabolism of nucleic acids) [86,87]. Rodríguez-Sauceda [88] reported that phenolic compounds sensitize the cell membrane, and when the sites of action become saturated, the cell suffers serious damage, causing the membrane to collapse.

Saleem and Saeed [76] analyzed the antibacterial activity of lemon peel water extract (5 mg/mL) against several bacterial strains including *Ps. aeruginosa, K. pneumoniae, Serratia marcescens, E. coli, Proteus vulgaris,* and *S. typhii*. They reported inhibition zone diameters of 32.0, 35.0, 28.0, 32.0. 32.0, and 30.0 mm. In a similar project, Otang and Afolayan [80] carried out a study to determine the antibacterial activity of lemon peel ethanol extract against a panel of bacterial strains implicated in skin diseases. These authors reported that at 50 mg/mL lemon peel showed inhibition zone diameters of 17.0, 17.0, 15.0, and 15.0 mm against *Enterococcus faecalis, Bacillus subtilis, Klebsiella pneumoniae*, and *Staphylococcus aureus*, respectively. Abdel-Salam and Fatma [81] reported that the inhibition zones diameters of ethanol extracts obtained from mandarin (*C. reticulata*) against several bacterial strains including *E. coli, S. aureus, Pseudomonas flourescens,* and *Ps. Aeruginosa* were 14.0, 13.0, 10.0, and 20.0 mm, respectively. More recently, Guo et al. [89] studied the antibacterial effect of the ethanol-acetone extracts from Newhall navel orange peel against *E. coli, B. subtilis, S. aureus*, and *Xanthomonas citri* subsp. *citri*. The authors found inhibition zones diameters at 100 mg/mL of 24.50, 30.36, 11.75, and 19.01 mm for *E. coli, B. subtilis, S. aureus*, and *X. citri*, respectively.

As mentioned previously, several extracts obtained from citrus peels also had demonstrated antifungal properties. Thus, Hernández et al. [90] investigated the antifungal capacity of orange peel extract against three relevant post-harvest fungal pathogens, *Monilinia fructicola*, *Botrytis cinerea*, and *Alternaria alternata*. These authors reported that at 24 h and 1 g/L of orange peel extracts, the inhibition of conidial germination was from 94.2, 82.1, and 46.5% for *A. alternata*, *M. fructicola*, and *B. cinerea*, respectively. Similarly, Saleem and Saeed [76] analyzed the antifungal activity of lemon peel water extract (5 mg/mL) against *Aspergillus niger* and *Penicillium citrinum*. They reported inhibition zones diameters of 16.0 and 15.0 mm for *A. niger* and *P. citrinum*, respectively. Olakunle et al. [91] analyzed the antifungal activity of orange peel aqueous extracts at 100 mg/mL for *A*. *niger* and *A. alternata*. The results obtained on day 3 showed that *A. niger* had an inhibition zone of 3.30 mm while the inhibition zone of *A. alternata* was 5.0 mm.

## 6. Techno-Functional Properties

Citrus co-products are exceptional sources of dietary fiber, which may be classified into soluble dietary fiber (SDF) and insoluble dietary fiber (IDF). SDF includes pectin, gums, and a part of hemicellulose, while IDF mostly includes cellulose, hemicellulose, and lignin [92]. Deng et al. [93] found that the total dietary fiber content of peels obtained from pummelo (*Citrus grandis* L. Osbeck) and grapefruit (*C. paradisi* Mcfad) cultivated in China were 16.0 and 22.0 g/100 g, respectively, with an IDF/SDF ratio of 2.06 and 2.9. In a similar study, Wang et al. [94] reported that the total dietary fiber of lemon (*C. lemon*), orange (*C. sinensis*), and mandarin (*C. reticulata*) peels were 64.07, 63.24, and 62.87 g/100 g, with an IDF/SDF ratio of 3.90, 3.63, and 3.81 for lemon, orange, and mandarin, respectively.

Due to this high content in dietary fiber, citrus co-products showed very interesting techno-functional properties such as the water holding capacity (WHC), oil holding capacity (OHC), swelling capacity (SC), foam capacity (FC), as well as emulsion capacity (EC) [95,96,97,98,99]. These properties are related with several factors including chemical structure of constituents, porosity, particle dimension, ionic form, extraction conditions, pH values, insoluble:soluble dietary fiber ratio, drying process, etc. [100]. In this sense, Lopez-Marcos et al. [95] analyzed the water holding capacity (WHC) and oil holding capacity (OHC) as well as the swelling capacity (SC) of lemon (*C. lemon*) and grapefruit (*C. paradise*) flours with a total dietary fiber content of 66.71 and 69.15 g/100 g, respectively, which were obtained from co-products of juice extraction. These authors reported values for WHC of 7.96 and 6.38 g water/g sample for OHC values of 1.69 and 2.30 g oil/g sample, while the values for swelling capacity were 5.69 and 6.50 mL/g for lemon and grapefruit flours, respectively. In a similar study, Huang et al. [96] analyzed the techno-functional properties of orange peel powder with a dietary fiber content of 64 g/100 g. They reported that the WHC and OHC values were 6.97 water/g sample and 2.94 g oil/g sample. Hosseini et al. [97] carried out a study to determine the WHC and OHC of pectin extracted from sour orange peel. They reported water holding capacity and oil holding capacity values of 3.10 and 1.32 g water or oil/g pectin. In another study, Saikia and Mahanta [98] investigated the WHC, OHC, and SC of mandarin (*C. reticulata*) powder extract that had a total dietary fiber of 37.82 g/100g. These authors reported values of 13.96 and 12.00 g water or oil/g while the SC value was 10.30 mL/g. Wang et al. [99] analyzed the emulsion capacity of orange peel and reported values of 56.40 mL/100 mL.

Hydration properties (WHC and SWC) of citrus co-products are related to their ability to retain water and decrease cooking losses. These properties play an important role in developing food texture, especially in comminuted and cooked meat products. In addition, low values of WHC or SWC in meat and meat products diminished visual appeal due to excess purge in packages and inferior palatability traits related to juiciness and tenderness. The emulsifying capacity of citric co-products is a highly valued property in the development of meat products since it facilitates the process of elaboration of cooked products such as frankfurters or mortadella by stabilizing these emulsions.

## 7. Biological Effects

Citrus co-products, due to the high content in their composition of bioactive compounds including flavonoids among others, have gained special attention by the scientific community because of their unique and enhanced therapeutic properties against different chronic diseases such as cancer, diabetes, and particularly cardiovascular diseases. Table 3 shows the health benefits of peel extracts obtained from peels of different *Citrus* species.

## 8. Addition of Bioactive Compounds from Citrus Co-Products to Meat and Meat Products

As mentioned above, two of the most used strategies for the development of healthier meat and meat products are acting on the animal feeding by incorporating in the diet products with a high content of bioactive compounds or acting on the meat products by adding these bioactive compounds as an ingredient of the formulation. A third way for the application of the bioactive compounds present in citrus co-products would be their use as active components in active packaging, coatings, and films.

### 8.1. Animal Feeding

The dietary supplementation with bioactive compounds with antioxidant activity seems to be a more effective way of delaying the protein and lipid oxidation of animal products and controlling stability. In this sense, a relatively high number of scientific works have examined the effects of citrus flavonoid-rich feed sources and crude citrus extracts on fresh meat stability (Table 4). As regards citrus crude extracts, Gravador et al. [116] found that the incorporation of dried citrus pulp in lamb diet significantly decreased protein radicals and carbonyls levels in fresh longissimus thoracis et lumborum. In a similar study, Luciano et al. [117] analyzed the effect of dietary whole dried citrus pulp on the antioxidant status of lamb tissues. These authors found that dried citrus pulp had no effect on the extent of lipid peroxidation in the small intestine, liver, plasma, and muscle. Nonetheless, when the lipid peroxidation was induced in muscle homogenates, the muscle from lambs fed dried citrus pulp presented lower lipid oxidation values. In a more recent study, Tayengwa et al. [118] observed that feeding beef with dried citrus pulp comprised of seeds, pulp, and peels reduced the thiobarbituric reactive substances and carbonyl contents of fresh longissimus lumborum.

Regarding citrus flavonoids, Iskender et al. [119] analyzed the effect on oxidative stability of naringin and hesperidin addition in laying hens feed. According to the authors, the addition of both citrus flavonoids decreased malondialdehyde concentration. In the same line, Simitzis et al. [120] carried out a study to examine the effects of citrus flavonoids hesperidin and naringin dietary supplementation (2.5 g/kg) on lamb antioxidant status and meat quality characteristics. These authors found that hesperidin and naringin dietary supplementation reduced plasma malondialdehyde levels at the end of study. In addition, both citrus flavonoids also reduced malondialdehyde levels in meat stored at 4 °C for up to 8 days.

### 8.2. Meat Products

As mentioned above, another of the strategies that can be used to prepare healthier meat products is the application, as ingredients, of either citrus extracts or citrus flours, which are rich in bioactive compounds. These citrus extracts or flours can be applied directly as a component of the formulation or can be used to replace any component with harmful effects on health. In addition, these extracts may have a positive effect on the oxidative and microbiological stability of the food, increasing its shelf life.

To develop healthier meat products, citrus co-products can be applied to enrich the dietary fiber content or to act as a fat replacer. In this sense, Soncu et al. [128] designed a study to analyze the effect of lemon fiber addition (2, 4, and 6%) on the cholesterol content of low-fat beef burgers. These authors found that lemon fiber addition decreases saturated fatty acids content and reduces the cholesterol content in a concentration-dependent manner. Similarly, Song et al. [129] elaborated low-fat Frankfurt sausages added with different concentrations of citrus fiber (1, 2, and 3%). These authors reported that sausage samples added with citrus fiber had a lower content of saturated fatty acids and improved water-binding ability. In more recent studies, Gedikoğlu and Clarke [130] analyzed the effect of different citrus fiber levels (0%, 1%, 5%, and 10%) addition on chemical composition of ground beef meatballs. They reported that all samples added with citrus fiber had lower saturated fatty acids content and higher dietary fiber content compared with a control sample. Almaráz-Buendia et al. [131] analyzed the addition of a water-oil nanoemulsion, formulated with orange essential oil and cactus acid fruit, as partial fat replacer in an emulsified meat system. According to the authors, the nanoemulsion addition decreased malonaldehyde production and reduced lipid oxidation while the bioactive compounds and antioxidant activities were incremented. Likewise, Selim et al. [132] analyzed the effect of orange peels addition, used as fat replacer, on oxidant stability of low-fat beef burger. According to these authors, the peroxide values of samples where orange peels were used as fat replacer were lower than a control sample with a reduction of 95.69 and 96.79% for a replace of 2.5, 5, and 7.5%, respectively. In a recent study, Silva et al. [133] evaluated the influence of the orange albedo flour as an animal fat substitute in beef burgers. According to the authors, the percentage of lipids was reduced by up to 70%; nevertheless, the water retention capacity and shrinkage rate were negatively affected.

With respect to improving the shelf life of meat and meat products, Sayari et al. [134] carried out a study to determine the lipid oxidation stability of turkey sausage added with grapefruit peel extracts during storage period. The authors indicated that at the initial day TBA values for all samples added with grapefruit peel were lower than those for the control, while at the end of the study (day 13) a reduction in the lipid oxidation of 73.45% was obtained in samples added with grapefruit peel with respect to the control sample. In another related experiment, Klangpetch et al. [135] analyzed the effect of Kaffir lime (Citrus hystrix DC.) peels, at 0.1, 0.5, and 1% on the shelf life of raw chicken wings drumettes stored at 4 °C during 14 days. These authors reported that at the end of the storage period lipid oxidation values increased significantly in control samples (2.5 mg MDA/kg sample), but in samples treated with kaffir lime peels, at all concentrations, lower values were obtained (between 1.5 and 1.8 mg MDA/kg sample). In the same sense, Mahmoud et al. [136] evaluated the lipid stability of beef burger elaborated with different concentrations (2.5, 5.0, 7.5, and 10.0%) of orange peel. The authors indicated that lipid oxidation was successfully blocked by orange peel powder with respect to the control sample. Thus, the lowest TBA value was obtained in burger with 10% (0.163 mg malonaldehyde/kg sample), while in the control sample TBA values of 0.303 mg malonaldehyde/kg sample were achieved. In a more recent study, Ibrahim et al. [137] investigated the efficacy of adding orange or lemon peels flours (1 and 2 %) on lipid stability of prepared ground beef patties during refrigerated storage at 4 °C for 15 days. They reported that at the end of the storage period control samples had TBA values of 1.73 mg malonaldehyde /kg sample while the patties added with 2% of orange or lemon peel flour showed TBA values of 0.92 and 0.91 mg malonaldehyde/kg sample. Interesting research was also conducted by Nishad et al. [138] who investigated the potential of grapefruit peel and the binary mixture grapefruit peel nutmeg extracts in controlling lipid oxidation of meat balls during frozen storage. These authors found that the extracts effectively retarded the formation of lipid peroxides and malondialdehydes as well as protein oxidation. Powell et al. [139] analyzed the effects of sodium tripolyphosphate replacement with citrus fiber on lipid oxidation content of pork Bologna sausage. These authors found that TBA values were lower than 0.2 mg/kg malondialdehyde throughout the storage period (98 days). Haque et al. [140] explored the effect of different levels of orange peel extract on lipid stability of beef muscle storage at −20 °C during 60 days. They reported that control fresh beef samples exhibited significantly higher TBARS values (0.79 mg/malonaldehyde/kg sample) at the end of the frozen storage time as compared to 0.2, 0.3, and 0.4% orange peel extract treated beef samples with values of 0.70, 0.69, and 0.59 mg/malonaldehyde/kg sample, respectively.

## 9. Conclusions

The high amount of co-products generated during the industrialization of citrus fruits, together with their content in bioactive compounds (polyphenols among others) with interesting functional properties, such as antioxidant and antibacterial properties, have promoted the development of processes for their valorization, contributing to the sustainability of this sector. The food industry is one of the sectors that is using different extracts obtained from citrus co-products, mainly due to pressure from consumers who demand new products with a lower content of synthetic preservatives and obtained through sustainable and eco-efficient processes; in this case, citrus co-products could be a viable alternative to develop healthier and sustainable products.

Regarding the meat industry, citrus co-products are being used for animal feed, contributing to increased raw meat stability, and also for meat products processing, as substitutes of additives with antioxidant or antibacterial properties or even as sources of bioactive compounds with interesting techno-functional properties (water retention, emulsion, or gelification capacity). In addition, they are used as a tool to reduce the amount of nitrites in the final product. In all these cases, their use is contributing to an increase in meat products shelf life and consumer healthiness. Nevertheless, it should be borne in mind that if the citrus co-products are added directly without any previous treatment, it could lead to the appearance of strange flavors or odors that would substantially modify the organoleptic properties of the product. One way to solve this problem would be by performing treatments to obtain a product as neutral as possible that does not provide flavor, aroma, or color. In addition, the concentration added will be the minimum to produce the beneficial effects and avoid the organoleptic changes. This is as long as it is to apply it as if it were an intermediate food product. Another important aspect to highlight is the possible allergenicity of citrus co-products. In these potential ingredients a certain concentration of proteins can be found that could potentially cause allergies, since allergenicity is a type-I hypersensitivity reaction caused by protein antigens found in various food sources, marked by elevated levels of IgE antibodies that can lead to potentially life-threatening clinical reactions. To avoid this, enzymatic treatments could be used to break down these proteins and thus obtain a less allergenic product.

Thus, taking advantage of citrus co-products as a source of bioactive compounds and their use as ingredient for several formulations in the food industry has become, today, a promising field. However, this process involves interdisciplinary research, which can engage in diversified fields, including food chemistry, food technology, biotechnology, molecular biology, or toxicology.

## Figures and Tables

**Table 1:** Antioxidant activity of peel extracts obtained from different *Citrus* species.

Source	Extraction Methodology	Antioxidant Activity	Ref.
Peel *Citrus reticulata*	0.25 g sample; solvent: methanol (80%), ratio (1/100 *w/v*); shake overnight; centrifuged at 5000× *g* for 10 min.	DPPH value of 21.92 mg vitamin C equivalent antioxidant capacity (VCEAC)/dry weight and average of ABTS value of 78.70 mg VCEAC/ dry weight in peel.	[57]
Peel *Citrus reticulata*	0.15 g sample; solvent: methanol (80%), ratio (5/100 *w/v*); mortar and pestle on an ice bath; centrifuged at 4500× *g* for 5 min	DPPH activity (A.U.) of 2.3; FRAP activity (A.U.) of 2.8	[58]
Peel *Citrus reticulata*	0.5 g sample; solvent: ethanol (70%), ratio (5/100 *w/v*); boiling during 60 min. Freeze-dried; centrifuged at 4500× *g* for 5 min	ABTS value of 3.22 mmol Trolox/100 g FW	[15]
Peel *Citrus sinensies*	5 g sample; solvent: methanol (67%), ratio (1/10 *w/v*); centrifuged at 3600 rpm for 15 min	Concentration of the peel extract necessary to inhibit the formation of DPPH radicals in 50% of 5.21 mg/mL	[59]
Peel *Citrus sinensies*	25 g sample; solvent: ethanol (100%), ratio (1/12 *w/v*); Soxhlet-extracted	Concentration of the peel extract necessary to inhibit the formation of DPPH radicals in 50% of 0.7 mg/mL while the β-carotene bleaching inhibition (50%) was 0.95 mg/mL	[60]
Peel *Citrus sinensies*	2 g sample; solvent: ethanol (75%), ratio (1/5 *w/v*); incubated at 65 °C for 40 min	Antioxidant values in DPPH assay of 3.7 mg Trolox Equivalent/g sample while in FRAP assay the antioxidant value was 155 mg Trolox Equivalent/g sample	[61]
Peel *Citrus lemon*	100 g sample; solvent: methanol (100%), ratio (1/10 *w/v*); Soxhlet-extracted for 60 min	Concentration of the peel extract necessary to inhibit the formation of DPPH radicals in 50% of 22.97 µg/mL	[62]
Peel *Citrus lemon*	1 g sample; solvent: water, ratio (1/20 *w/v*); ultrasonic bath (40 kHz, 150 W) for 20 min	The DPPH free radical scavenging activity of 49.29 % while in FRAP assay the antioxidant value was 155 µMol ferrous sulfate Equivalent/L sample	[63]
Peel *Citrus lemon*	2 g sample; solvent: methanol (80%), ratio (1/7.5 *w/v*); ultrasonic bath (40 kHz, 300 W) for 30 min	Antioxidant values in DPPH assay ranging from 8.28 to 16.49 µmol Trolox Equivalent/g sample while in ABTS assay the antioxidant values varied from 25.21 to 38.58 mg Trolox Equivalent/g sample	[64]
Peel *Citrus paradisi*	1 g sample (flavedo or albedo); solvent: ethanol (70%), ratio (1/10 *w/v*); ultrasonic bath (40 kHz, 300 W) for 30 min	Antioxidant values in DPPH assay of 0.50 and 0.62 mg Trolox Equivalent/g sample for flavedo and albedo, respectively while in ABTS assay the antioxidant values were 1.22 and 3.10 mg Trolox Equivalent/g sample for flavedo and albedo, respectively.	[65]
Peel *Citrus paradisi*	5 g sample (white and pink grapefruit); solvent: methanol (100%), ratio (1/4.4 *w/v*); accelerated solvent extractor ASE 200	Antioxidant values in DPPH assay of 32.46 and 25.18 mg Trolox Equivalent/g sample for white and pink grapefruit, respectively while in ABTS assay the antioxidant values were 122.34 and 99.46 mg Trolox Equivalent/g sample for flavedo and albedo, respectively.	[66]

**Table 2:** Antibacterial activity of peel extracts obtained from different *Citrus* species.

Source	Methodology/ Concentration	Bacterial Strains	Antimicrobial Effect	Ref.
Blood orange peel (*Citrus sinensis*)	Disc diffusion assay 10 mg/mL	*Micrococcus luteus*, *S. aureus*, *Bacillus cereus*, *Pseudomonas aeruginosa*, *Salmonella enterica*, *Listeria monocytogenes*, and *Enterobacter* sp.	Inhibition zones diameters (mm) of 34.0, 36.0, 32.0, 19.0, 39.5, 33.5, and 30.0, respectively	[58]
Bergamot peel (*Citrus bergamia*)	Minimum inhibitory concentration	*S. aureus*; *Ps. aeruginosa*; *L. monocytogenes*; *Escherichia coli*	Minimal ihhibitory concentration of 10.0, 8.0, 8.0, and 9.0 mg/mL, respectively	[72]
Mandarin peel (*Citrus reticulata*)	Disc diffusion assay 0.50 mg/mL	*Bacillus subtilis, K. pneumonia*	Inhibition zones diameters (mm) of 17.25 and 15.76, respectively	[73]
Orange peel (*Citrus sinensis*)	Disc diffusion assay 0.50 mL/mL	*S. aureus* and *E. coli*	Inhibition zones diameters (mm) of 16.0 and 10.0, respectively	[74]
Orange peel (*Citrus reticulata* var. Kinnow)	Minimum inhibitory concentration	*S. aureus*, *Salmonella typhii*, *K. pneumonia*, and *E. coli*	Minimal ihhibitory concentration of 781.25, 1562.5, 3125, and 3125 µg/mL, respectively	[75]
Orange peel (*Citrus sinensis*)	Disc diffusion assay 5 mg/mL	*Ps. aeruginosa*, *K. pneumoniae*, *Serratia marcescens*, *E. coli*, *Proteus vulgaris*, and *S. typhii*	Inhibition zones diameters (mm) of 25.0, 29.0, 18.0, 19.0, 18.0, and 16.0, respectively	[76]
Lemon peel (*Citrus limon* Osbeck)	Disc diffusion assay 5 mg/mL	*S. aureus*, *E. faecalis*, *Aeromonas hydrophila*, *Streptococcus pyogenes*, *L. monocytogenes*, and *Lactobacillus casei.*	Inhibition zones diameters (mm) of 25.0, 35.0, 28.0, 32.0, 28.0, and 25.0, respectively	[76]
Bergamot peel (*Citrus bergamia*)	Minimum inhibitory concentration	*E. coli*, *Pseudomonas putida*, *Salmonella enterica*, and *B. subtilis,*	Minimal ihhibitory concentration of 400, 800, 800, and 1000 µg/mL, respectively	[77]
Lemon peel (*Citrus limon* Osbeck),	Disc diffusion assay 0.10 mL/mL	*S. aureus* and *E. coli*	Inhibition zones diameters (mm) of 20.6 and 19.50, respectively	[78]
Lemon peel (*Citrus limon*)	Disc diffusion assay 100 µg/mL	*S. aureus*, *K. pneumoniae*, *Shigella flexneri* and *E. coli*	Inhibition zones diameters (mm) of 14.0, 14.0, 21.0, and 16.0, respectively	[79]
Lemon peel (*Citrus limon*)	Disc diffusion assay 50 mg/mL	*Salmonella typhimurium*, *E. Coli*, *Ps. aeruginosa*, *Shigella sonnei* and *S. flexneri*	Inhibition zones diameters (mm) of 20.0, 15.0, 15.0, 17.0, and 15.0, respectively	[80]
Orange peel (*Citrus sinensis*)	Disc diffusion assay 50 mg/mL	*E. coli*, *S. aureus*, *Ps. flourescens*,and *Ps. aeruginosa*	Inhibition zones diameters (mm) of 11.0, 11.0, 10.0, and 25.0, respectively	[81]
Orange peel (*Citrus sinensis*)	Disc diffusion assay 0.2 mg/mL	*Candida albicans*, *A. niger* and *Penicillium notatum*	Inhibition zones diameters (mm) of 18.0, 2.0, and 2.0, respectively	[82]
Lemon peel (*Citrus limon*)	% growth inhibition 14 and 7 mg/mL	*A. alternata*	Diameter growth 21.52 and 28.96 mm	[83]
Mandarin peel (*Citrus reticulata*)	Disc diffusion assay 100 mg/mL	*Colletotrichum* spp., *P. digitatum*, *Curvularia* spp., and *Trichophyton mentagrophytes*	Inhibition zones diameters (mm) of 6.0, 12.0, 18.0, and 15.0, respectively	[84]

**Table 3:** Health benefits of peel extracts obtained from different *Citrus* species.

Activity	Source	Compounds or Extract	Effect	Ref.
Cardio protective effect	Peel *Citrus Changshan-huyou*	Flavononids extract (50, 100 mg/kg)	Significant reduction in serum contents of cholesterol, triglycerides, and low-density lipoprotein cholesterol	[101]
	Peel *Citrus reticulata*	Aqueous extracts rich in flavanones, glycosides flavone and methoxyflavones	Significant decrease in cholesterol, triacylglyceride, and glucose	[102]
	Peel *Citrus unshiu*	Peel extracts at 1 g/mL	Total serum cholesterol, triglycerides, and low-density lipoprotein cholesterol were significantly decreased	[103]
	Peel *Citrus grandis*	Ethanolic extracts rich in flavanones, glycosides flavone and Polymethoxyflavones	Extracts blocked the body weight gain, lowered fasting blood glucose, total serum cholesterol, liver lipid levels, and improved glucose tolerance and insulin resistance, and lowered serum insulin levels	[104]
	Peel *Citrus lemon*	Hydro-methanolic extracts with high flavonoid content	Declining trend for total cholesterol was obtained. Likewise, levels of low density lipoproteins and triglycerides were also reduced	[105]
Anti-inflammatory	Peel *Citrus reticulata*	High content of polymethoxylated flavones	The extract showed higher nitric oxide, inducible nitric oxide synthase (iNOS), and cyclooxygenase (COX-2) inhibitory activity	[51]
	Peel *Citrus sinensis*	Methanolic extract 75 mg/kg body weight	Significant down-regulation of COX-2, intercellular adhesion molecule -1, and tumor necrosis factor alfa in epididymal adipose tissue	[106]
	Peel *Citrus grandis*	Methanolic extract 300 and 500 mg/kg body weight	Inhibition of paw edema by 34.47% and 38.68%, respectively, relative to the controls.	[107]
Anti-diabetic	Peel *Citrus lemon*	Hexane extract at 10 mg/kg body weight	Decreased the blood glucose comparable to that of glimepiride. The peel extract stimulated β-cell of islets of Langerhans to secrete insulin and decreased the blood glucose level.	[108]
	Peel *Citrus sinensis*	Methanolic extract 50 and 100 mg/kg body weight	Reduction of fasting blood glucose (56.1% and 55.7%, respectively) and plasma insulin levels (22.9% and 32.7%, respectively) compared with untreated control samples	[109]
	Peel *Citrus reticulata*	Ethanolic extract 100 mg/kg body weight	Sgnificantly ameliorated the impaired oral glucose tolerance; the elevated serum fructosamine level; the diminished serum insulin and decreased liver glycogen content.	[110]
Anti-cancer	Peel *Citrus reticulata*	Polymethoxyflavones	Strong antiproliferative activity against human breast cancer (MCF-7), human lung carcinoma (A549), and human liver hepatoblastoma (HepG2) cell lines	[111]
	Peel *Citrus reticulata*	Hydro-ethanolic extracts rich in flavanones, glycosides flavone and Polymethoxyflavones	Reduction in human breast carcinoma (BT-474), human colon adenocarcinoma (Caco-2), and human liver hepatoblastoma (HepG2) cell lines viability	[15]
	Peel *Citrus sinensis*	Aqueous peel extracts and naringin	In vivo experiments revealed that the use of doxorubicin simultaneously with orange peel or naringin can reduce the tumor size	[112]
	Peel *Citrus sinensis*	Aqueous peel extracts contained 30% polymethoxyflavones, included tangeretin nobiletin and sinensitin	The development of tumors markedly decreased, with multiplicity decreasing 49% in the small intestine and 38% in the colon and induced apoptosis	[113]
	Peel *Citrus junos*	Ethanol extract rich in flavonoids at 100mg/kg/day	Significantly reduced the size of colorectal adenocarcinoma HT-29 tumor cells through reducing COX-2 expression in xenograft mice	[114]
	Peel *Citrus grandis*	ethanol–water extracts (0 to 100 mg/mL)	Antiproliferative effects in four cancer cell lines including A549 (human lung cancercell line), MCF-7 (human breast cancer cell line), HepG2 (human hepatoma cell line), and HT-29 (human colon cancer cell line).	[115]

**Table 4:** Effects of citrus flavonoid-rich feed sources and crude citrus extracts on fresh meat stability.

Animal (Muscle)	Product	Treatment	Effect on Meat	Ref.
Lamb (*longissimus thoracis et lumborum*)	Dried citrus pulp	24% or 35% dried citrus pulp	Citrus pulp significantly decreased protein radicals and carbonyls, and preserved more thiols within six days of storage compared to the control group.	[116]
Beef (*longissimus lumborum*)	Dried citrus pulp (seeds, pulp, peels)	150 g/kg for 90 days	Beef antioxidant activity was higher than control sample. Beef thiobarbituric reactive substances and carbonyl contents were lower than the control sample	[118]
White laying hens	Citrus naringin or hesperidin	0.5 g/kg diet during eight weeks	The treatment decreased malondialdehyde concentration as well as increased glutathione reductase, glutathione peroxidase, glutathione-S-transferase, and superoxide dismutase	[119]
Lamb (*longissimus thoracis*)	Citrus naringin or hesperidin	2.5 g/kg during 35 days	Both flavonoid and vitamin E dietary supplementation reduced blood plasma MDA levels	[120]
Beef (*longissimus thoracis*)	Dried citrus pulp	2.5 g/kg dry matter of feed for 90 days	Treatment did not influence the antioxidant status. Increased the proportion of conjugated linoleic acids and polyunsaturated fatty acids in beef	[121]
Pork (*longissimus dorsi*)	Citrus naringin	1.5 g kg dry matter of feed for 50 days	Naringin significantly increased superoxide dismutase activity and total anti-oxidative capacity in meat	[122]
Broiler breast meat	Citrus naringenin	5, 10 and 20 mg/kg for 42 days	Malondialdehyde values decreased in tissue samples in a dose-dependent manner	[123]
Broiler breast and thigh meat	Citrus naringin or hesperidin	0.75 or 1.5 g/kg for 42 days	Malondialdehyde values decreased in tissue samples in a dose-dependent manner	[124]
Broilerbreast muscle and liver	Citrus hesperidin	20 mg per kg of feed for 42 days	Improved the hepatic and muscle antioxidant and superoxide dismutase activities. Decreased the hepatic malondialdehyde concentration and muscle fat by the treatments.	[125]
Japanese quails breast meat	Orange peel extract	100 or 200 mg/kg	Decreased malondialdehyde levels on liver and heart tissues. Increased Glutathione peroxidase activity and glutathione production in liver and heart tissues	[126]
Rabbit	Dry lemon	1% or 2% in their daily diet	Increased enzymatic and non-enzymatic antioxidant activities superoxide dismutase, catalase, glutathione-S-transferase, glutathione peroxidase, and malondialdehyde in serum and liver tissues.	[127]

## Data Availability

The data presented in this study are available on request from the corresponding author.
